# Comparison of efficacy and safety between VKAs and DOACs in patients with atrial fibrillation after transcatheter aortic valve replacement: A systematic review and meta‐analysis

**DOI:** 10.1002/clc.23909

**Published:** 2022-08-28

**Authors:** Jie Yan, Ming Liu, Yu Zhang, Danning Yang, Fengshuang An

**Affiliations:** ^1^ The Key Laboratory of Cardiovascular Remodeling and Function Research, Chinese Ministry of Education, Chinese National Health Commission and Chinese Academy of Medical Sciences, The State and Shandong Province Joint Key Laboratory of Translational Cardiovascular Medicine, Department of Cardiology Qilu Hospital of Shandong University Jinan China

**Keywords:** anticoagulation, atrial fibrillation, DOACs, TAVR, VKAs

## Abstract

In the past decade, direct oral anticoagulants (DOACs) have proven to be the best option for patients with nonvalvular atrial fibrillation. Nevertheless, evidence for the use of DOACs for anticoagulation in valvular atrial fibrillation, particularly after aortic valve replacement, remains inadequate. Thus, we conducted a meta‐analysis to compare the efficacy and safety of vitamin K antagonists (VKAs) and DOACs in patients with atrial fibrillation after transcatheter aortic valve replacement (TAVR). We conducted a comprehensive search of online databases, and 11 studies were included in the final analysis. The primary endpoint was all‐cause mortality. Secondary endpoints included stroke and cardiovascular death. The safe endpoint is major and/or life‐threatening bleeding. Subgroup analysis was conducted according to the different follow‐up time of each study. Random‐effects models were used for all outcomes. Statistical heterogeneity was assessed using *χ* ^2^ tests and quantified using *I* ^2^ statistics. Patients in the DOACs group had a significantly lower risk of all‐cause mortality compared with patients in the VKAs group (relative risk [RR]: 1.20, 95% confidence interval [CI]: 1.01–1.43, *p* = .04). This benefit may be greater with longer follow‐up. In a subgroup analysis based on the length of follow‐up, a significantly lower risk of all‐cause mortality was found in the DOACs group in the subgroup with a follow‐up time of >12 months (RR: 1.50, 95% CI: 1.07–2.09, *p* = .001). There were no significant differences between the two groups in cardiovascular death, stroke, and major and/or life‐threatening bleeding. For patients with atrial fibrillation after TAVR, the use of DOACs may be superior to VKAs, and the benefit may be greater with longer follow‐up. The anticoagulant strategy for atrial fibrillation after TAVR is a valuable direction for future research.

## 1 INTRODUCTION

As the population ages, the incidence, prevalence, and mortality of aortic valve disease, particularly calcific aortic valve disease, has risen substantially, contributing significantly to the disease burden among the elderly.^1^, ^2^ Patients with mild aortic stenosis (AS) may remain asymptomatic for many years.^3^ Once severe aortic stenosis develops, the symptoms and condition deteriorate, and conservative medical treatment tends to have a poor prognosis, quality of life, and long‐term survival unless surgery or intervention is performed.^4^, ^5^ Surgical aortic valve replacement (SAVR) is a traditional treatment for AS, but it is characterized by high surgical trauma and high surgical risk. Structural cardiac interventions have advanced by leaps and bounds in recent years, with transcatheter aortic valve replacement (TAVR) emerging as an alternative treatment for patients with symptomatic AS, inoperable aortic valve stenosis, or high risk of SAVR.^6^ With the gradual maturity of TAVR therapy, the therapeutic effect has been significantly improved and the indications are becoming more and more extensive. For symptomatic severe AS patients with low surgical risk, there was no significant difference in all‐cause mortality, stroke or myocardial infarction, or prosthetic valve failure in patients after TAVR compared to those who received SAVR.^7^

Prior and new atrial fibrillation (AF) is common in patients with severe AS receiving TAVR, and AF is associated with increased mortality and adverse ischemic and bleeding events.^8^ Warfarin is the main anticoagulant therapy for stroke prevention in patients with AF, but it has disadvantages such as narrow treatment window, variable dose–response, interaction with drugs and food, and the need for international normalized ratio (INR) detection.^9^, ^10^ Randomized clinical trials have demonstrated that DOAC is as good or better than warfarin for antithrombotic therapy in patients with AF.^11^, ^12^, ^13^, ^14^ The guidelines recommend DOACs as the first choice for anticoagulant therapy in patients with DOACs indications.^15^ DOACs are increasingly being used in place of warfarin, but the evidence for their effectiveness and safety in patients with valvular AF remains limited.^9^ The 2021 ESC/EACTS (European Society of Cardiology/European Association for Cardio‐Thoracic Surgery) guidelines for the management of valvular heart disease recommend that patients with TAVR without anticoagulant indications receive single antiplatelet drug therapy for life after 3–6 months of dual antiplatelet therapy (Class I recommendation, Level A evidence). In addition, lifetime anticoagulant therapy is recommended for patients with anticoagulant indications (Class I recommendation, Level B evidence).^16^ Due to the scarcity of data on patients after TAVR, no strong anticoagulation recommendations have been made so far. The ATLANTIS (Alteplase Thrombolysis for Acute Noninterventional Therapy in Ischemic Stroke) trial failed to demonstrate the superiority of full‐dose apixaban over the current standard of treatment in patients with or without indications for oral anticoagulants, apixaban was not different from standard care for the primary endpoint of death, stroke, myocardial infarction, systemic embolism, intracardiac or valvular thrombosis, deep vein thrombosis/pulmonary embolism, or major bleeding.^17^ The ENVISAGE‐TAVI AF (Edoxaban Compared to Standard Care After Heart Valve Replacement Using a Catheter in Patients With Atrial Fibrillation) trial evaluated the efficacy and safety of edoxaban and VKAs in AF patients after TAVR. The results showed that edoxaban was not inferior to VKA in terms of combined adverse events, but increased the risk of major bleeding.^18^

Compared with VKAs, the effect of DOACs on AF after TAVR has not been thoroughly studied, so we conducted a meta‐analysis to compare the efficacy and safety of VKAs and DOACs in patients with AF after TAVR.

## 2 MATERIALS AND METHODS

### 2.1 Data sources and study search strategy

We conducted a comprehensive search of studies comparing the efficacy and safety of VKAs or DOACs in patients with AF after TAVR in PubMed, Cochrane Central Register of Controlled Trials (CENTRAL), and ClinicalTrials.gov websites. The search term included “transcatheter aortic valve implantation or transcatheter aortic valve replacement, or TAVI or TAVR; AF; anticoagulation or anticoagulant or antithrombotic or vitamin K antagonist or VKA or Coumadin or Warfarin or novel oral anticoagulant or NOAC or direct oral anticoagulant or DOAC or Dabigatran or Apixaban or Rivaroxaban or Edoxaban”.

### 2.2 Inclusion and exclusion criteria

Studies were included in our meta‐analysis when the following criteria were met: (1) The study is limited to English and human subjects. (2) The study compared the efficacy or safety of VKAs and DOACs in patients with AF after TAVR. (3) At least one of these outcomes was reported in the study: all‐cause mortality, death from cardiovascular causes, stroke, and major and/or life‐threatening bleeding.

### 2.3 Data extraction and endpoints

All data were independently extracted from text, tables, and graphs by two authors (J. Y. and M. L.). Disagreements among reviewers were resolved through discussion to reach a consensus. The primary endpoint of this meta‐analysis was all‐cause mortality (death from all causes). Secondary endpoints were stroke (ischemic stroke) and cardiovascular death (death from cardiovascular causes). The safety endpoint is major and/or life‐threatening bleeding.

### 2.4 Methodological quality

Two investigators independently assessed the methodological quality of the included studies. The quality of randomized controlled trial (RCT), controlled clinical trial (CCT), and cohort studies was assessed using the Cochrane Risk of Bias Collaboration Tool,^19^ the Nonrandom Research Methodology Index (MINORS),^20^ and the Newcastle–Ottawa Scale (NOS).^21^ The comprehensive effect used relative risk (RR), with a confidence interval (CI) of 95%. A two‐sided *p* value was used, and *p* < .05 was considered significant. Random‐effects models were used for all outcomes. Statistical heterogeneity was assessed using the chi‐squared test (*p* < .10 was considered statistically significant for heterogeneity) and was quantified using the *I* ^2^ statistic. Subgroup analysis by follow‐up time was performed to further analyze the statistical results and explore possible sources of heterogeneity. The publication bias was tested by Egger regression to test the asymmetry of the funnel chart. We conducted sensitivity analysis by eliminating each included study one by one, looking for potential sources of heterogeneity. The above data were analyzed using Review Manager (RevMan version 5.4.1) and Stata (16.0) software.

## 3 RESULTS

**Figure 1:**
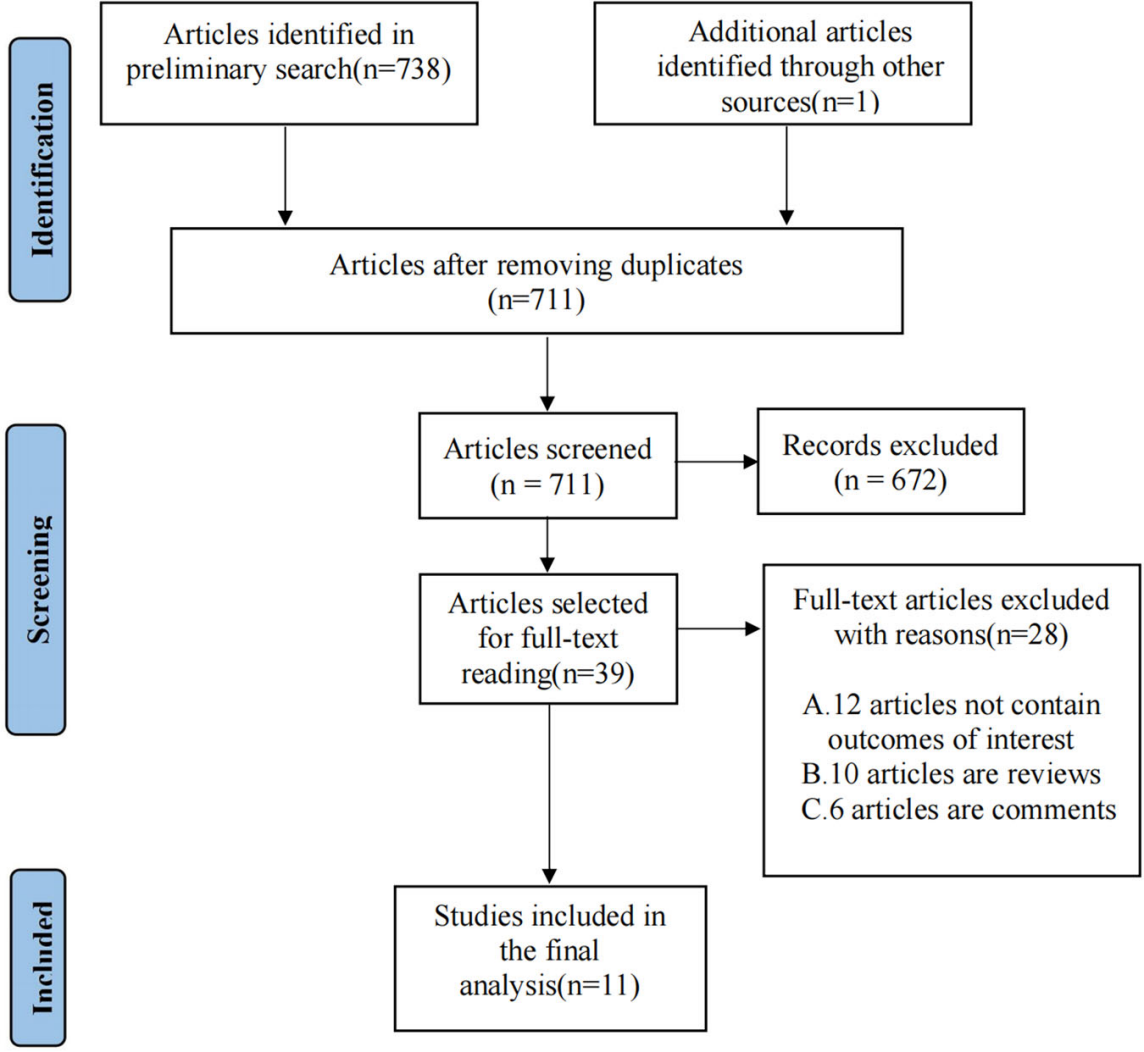
Flow diagram for the study search process

The literature retrieval and screening process are shown in Figure 1. According to the search strategy, a total of 738 articles and 1 trial from the American College of Cardiology Annual Scientific Session were searched and recorded. Twenty‐eight duplicate articles were removed. After scanning the titles and abstracts, 672 irrelevant reports were excluded. Read the full text of the remaining 39 records. Finally, a total of 39 full‐text articles were read, and 11 studies^17^, ^18^, ^22^, ^23^, ^24^, ^25^, ^26^, ^27^, ^28^, ^29^, ^30^ met the eligibility criteria.

The main characteristics of the included studies and population are shown in Table 1. The RCTs were evaluated as high quality (Supporting Information: Figure 1), the CCT had a global ideal score being 21 (>16) (Supporting Information: Table 1), and all cohort studies were considered of high quality because of the scores ranging from 7 to 9, with an average of 7.875 (Supporting Information: Table 2). We evaluated the publication bias of the outcome indicators with a funnel plot, and the results are shown in Supporting Information: Figure 2.

**Table 1:** Characteristics of the included trials

Study	Year	Total patients	No of patients (VKAs/DOACs)	Follow‐up (months)	Age (years)	Body mass index (kg/m^2^)	Female	Hypertension	Diabetes mellitus	CHA2DS2‐VASc score	HAS‐BLED score
Kosmidou et al.	2019	933	778/155	24	82.8 ± 6.7	28.4 ± 6.1	34.4 (321/933)	91.7 (856/933)	35.3 (329/933)	5.6 ± 1.3	NA
Seeger et al.	2017	272	131/141	12	81.3 ± 5.9	27.1 ± 4.7	40.5 (134/272)	NA	32.4 (88/272)	4.9 ± 1.2	3.1 ± 1.1
Kalogeras et al.	2019	217	102/115	24	82.2 ± 6.1	26.6 ± 5.8	58.5 (137/217)	NA	25.3 (55/217)	NA	NA
Tanawuttiwat et al.	2022	21 131	13 004/8127	12	83.6 ± 6.7	25.7 ± 6.0	43.3 (9149/21131）	91.7 (19368/21131)	36.7 (7788/21131)	3 ± 1.5	NA
Geis et al.	2018	326	172/154	6	83.0 ± 5.1	26.8 ± 5.3	52.8 (172/326)	93.5 (305/326）	32 (104/326）	4.7 ± 1.3	2.8 ± 1.1
OCEAN	2020	403	176/227	18.3	84.4 ± 4.7	22.2 ± 3.8	66.7 (269/403)	76.2 (307/403)	24.3 (98/403)	5.1 ± 1.1	2.7 ± 0.8
Jochheim et al.	2019	962	636/326	12	81.3 ± 6.3	26.5 ± 5.0	52.5 (505/962)	89.6 (862/962)	32.23 (311/962)	NA	NA
Butt et al.	2021	735	516/219	27	82.3 ± 5.7	NA	46.3 (340/735）	88.2 (648/735)	22.3 (164/735)	4.9 ± 1.3	3.3 ± 1
Mangner et al.	2019	299	117/182	12	80.0 ± 4.8	27.7 ± 5.2	54.8 (164/299)	97.3 (291/299)	55.0 (126/299）	5.0 ± 0.7	3.0 ± 0.7
ENVISAGE‐TAVI AF	2021	1426	713/713	36	82.1 ± 5.4	27.7 ± 5.6	47.5 (678/1426)	93.3 (1331/1426)	40.0 (527/1426)	4.5 ± 1.4	NA
ATLANTIS	2021	451	228/223	12	82.0 ± 6.3	27.4 ± 5.3	NA	NA	NA	4.45 ± 1.4	NA

Abbreviations: ATLANTIS, Alteplase Thrombolysis for Acute Noninterventional Therapy in Ischemic Stroke; CHA_2_DS_2_‐VASc, congestive heart failure, hypertension, age, diabetes mellitus, prior stroke or TIA or thromboembolism, vascular disease, age, sex category; DOAC, direct oral anticoagulant; ENVISAGE‐TAVI AF, Edoxaban Compared to Standard Care After Heart Valve Replacement Using a Catheter in Patients With Atrial Fibrillation; OCEAN, Olpasiran Trials of Cardiovascular Events And LipoproteiN(a) Reduction; VKA, vitamin K antagonists.

### 3.1 Primary endpoint

**Figure 2:**
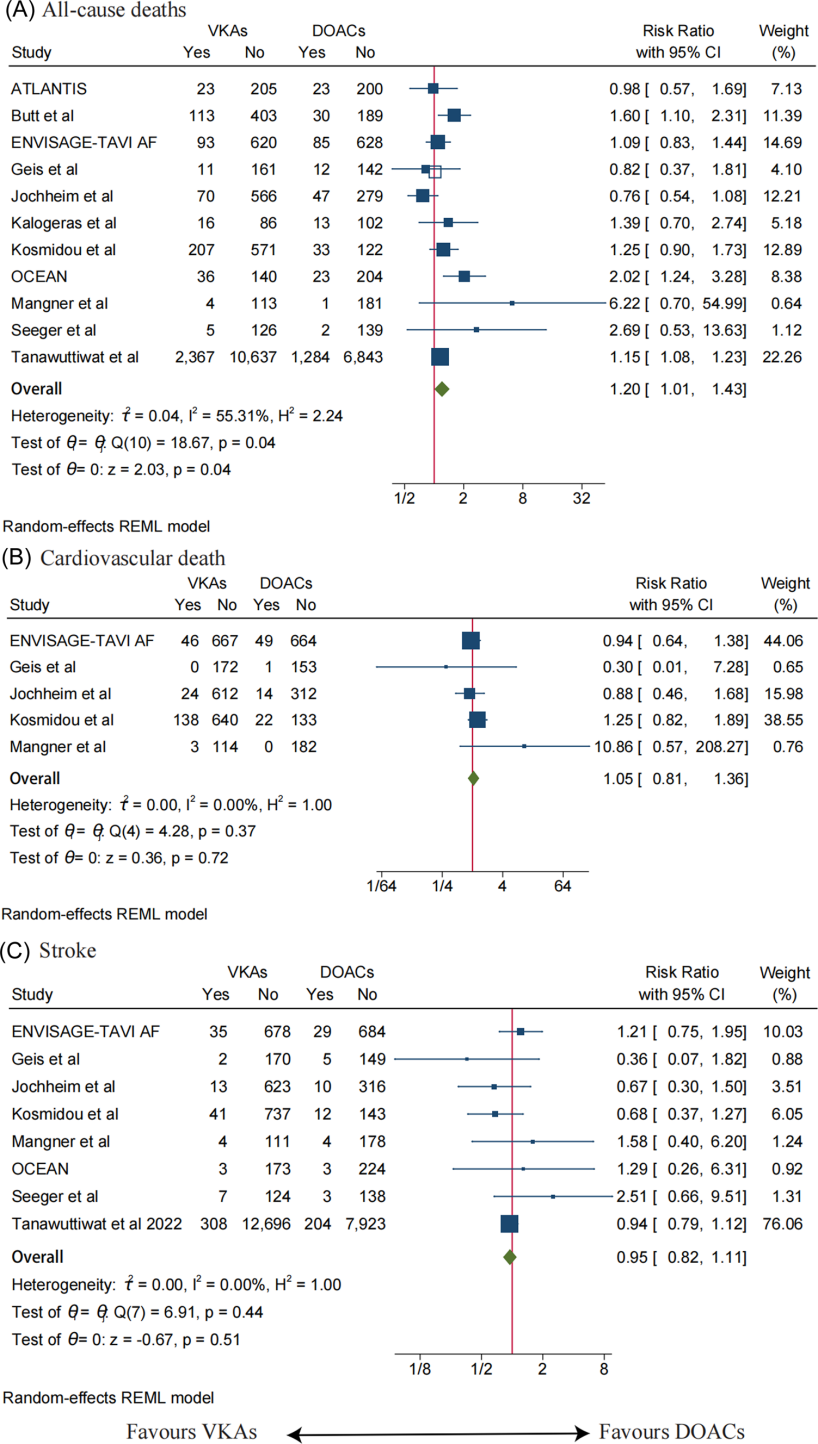
(A) Meta‐analysis for the risk of all‐cause death. (B) Meta‐analysis for the risk of cardiovascular death. (C) Meta‐analysis for the risk of stroke. The size of the box is proportional to the weight of the study in the meta‐analysis. ATLANTIS, Alteplase Thrombolysis for Acute Noninterventional Therapy in Ischemic Stroke; CI, confidence interval; DOACs, direct oral anticoagulants; ENVISAGE‐TAVI AF, Edoxaban Compared to Standard Care After Heart Valve Replacement Using a Catheter in Patients With Atrial Fibrillation; OCEAN, Olpasiran Trials of Cardiovascular Events And LipoproteiN(a) Reduction; RR, risk ratio; VKAs, vitamin K antagonists.

A total of 11 studies reported all‐cause deaths, and pooled analysis showed statistically significant differences between the VKAs and DOACs groups (RR: 1.20, 95% CI: 1.01–1.43, *p* = .04) (Figure 2A), with moderate heterogeneity (*I* ^2^ = 55.31%, *p* = .045). Sensitivity analysis was conducted by excluding each study item by item and the results remained stable after removing each study individually.

### 3.2 Secondary endpoints

Five studies reported cardiovascular death, and meta‐analysis demonstrated no statistical difference in cardiovascular death between patients in the VKAs group and those in the DOACs group (RR: 1.05, 95% CI: 0.81–1.36, *p* = .72) (Figure 2B), with no statistical heterogeneity (*I* ^2^ = 0.00%, *p* = .369). When each study was excluded, the results remained stable.

Eight studies reported the occurrence of stroke in patients, and pooled analysis showed no statistical difference between patients with VKAs and patients with DOACs (RR: 0.95, 95% CI: 0.82–1.11, *p* = .51) (Figure 2C), and no statistical heterogeneity between studies (*I* ^2^ = 0.00%, *p* = .439). By excluding each study for sensitivity analysis, the results remained stable.

### 3.3 Safety endpoint

**Figure 3:**
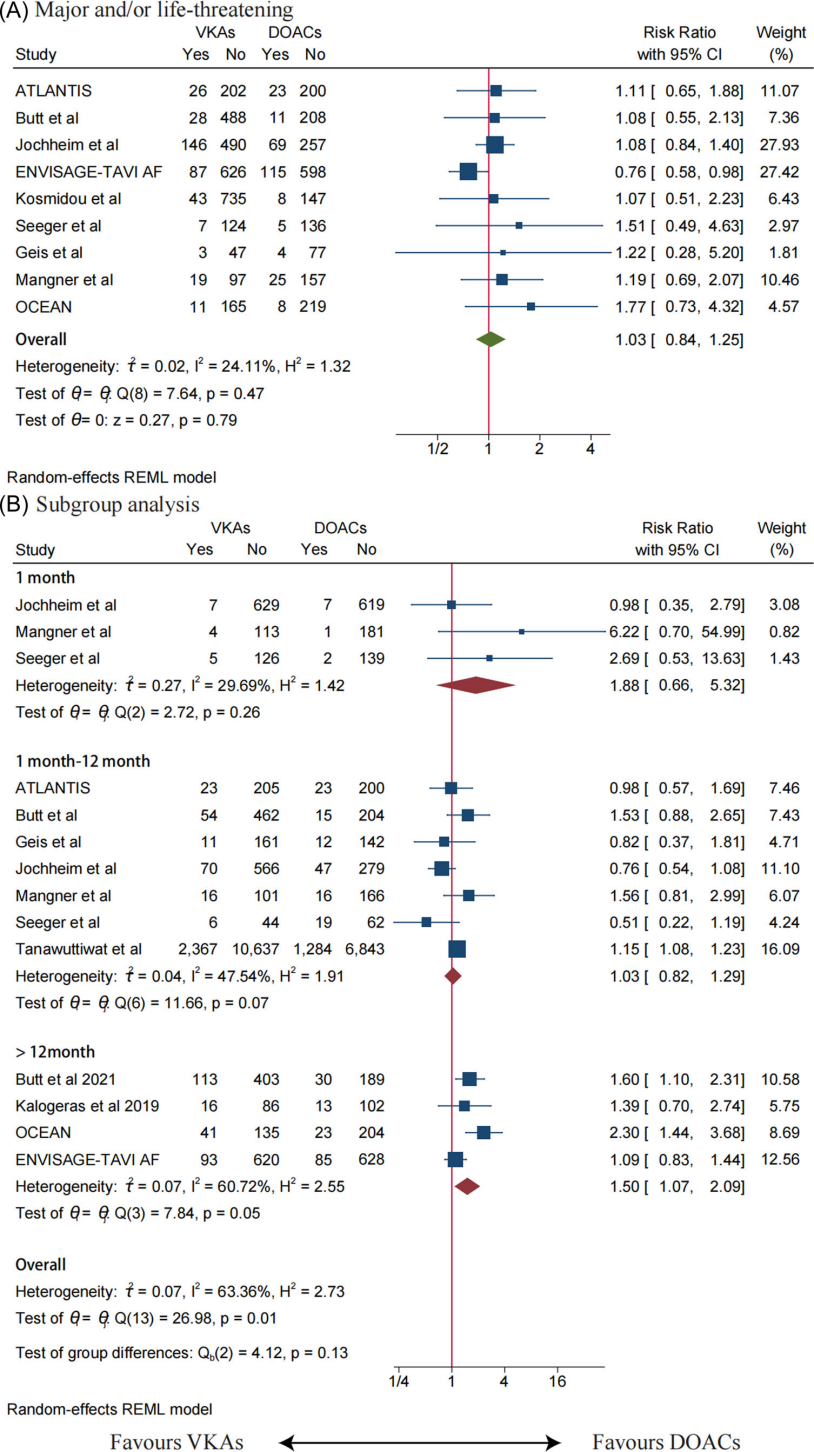
(A) Meta‐analysis for the risk of major and/or life‐threatening. (B) Subgroup analysis based on follow‐up time. The size of the box is proportional to the weight of the study in the meta‐analysis. ATLANTIS, Alteplase Thrombolysis for Acute Noninterventional Therapy in Ischemic Stroke; CI, confidence interval; DOACs, direct oral anticoagulants; ENVISAGE‐TAVI AF, Edoxaban Compared to Standard Care After Heart Valve Replacement Using a Catheter in Patients With Atrial Fibrillation; OCEAN, Olpasiran Trials of Cardiovascular Events And LipoproteiN(a) Reduction; RR, risk ratio; VKAs, vitamin K antagonists.

There was no significant difference in major and/or life‐threatening between the VKAs and DOACs groups (RR: 1.03, 95% CI: 0.84–1.25, *p* = .79) (Figure 3A), with mild statistical heterogeneity (*I* ^2^ = 24.11%, *p* = .469). After excluding each study, the results remained stable.

### 3.4 Subgroup analysis

Due to moderate heterogeneity in the all‐cause death analysis, a subgroup analysis was performed. The 1‐ and 12‐month subgroup of patients with AF after TAVR showed no statistical difference in all‐cause mortality between patients using VKAs and DOACs. However, follow‐up over 12 months subgroup showed statistically significant differences between the VKAs and DOACs groups, with patients in the DOACs group having a significantly lower risk of all‐cause mortality (RR: 1.50, 95% CI: 1.07–2.09, *p* = .001) (Figure 3B). There was significant heterogeneity in the result (*I* ^2^ = 60.72%, *p* = .05).

## 4 DISCUSSION

Our study shows that anticoagulant therapy with DOACs lowers the risk of all‐cause death in patients with AF after TAVR compared with VKAs. There was no significant difference between DOACs and VKAs in cardiovascular death, stroke, and major and/or life‐threatening bleeding. Our results provide a new clinical idea for choosing DOACs for patients with AF after TAVR.

The selection of antithrombotic strategies after TAVR is one of the hotspots of current research. ESC/EACTS have jointly published the 2021 ESC/EACTS Guidelines for the Management of Valvular Heart Disease, which recommends that patients without oral anticoagulant indications after TAVR should be given lifelong single‐drug antiplatelet therapy, and patients with oral anticoagulant indications should be given lifelong oral anticoagulant therapy.^16^ TAVR is mainly applied to elderly high‐risk patients, who are not only at high risk of thrombosis but also at high risk of bleeding. The incidence of thrombosis and bleeding events is closely related to the prognosis, and postoperative ischemic and hemorrhagic complications are very common.^31^ Many patients have AF before TAVR, which may be related to the cardiovascular pathophysiological conditions of elderly patients, such as atrial fibrosis and left atrial diameter enlargement.^32^ Meanwhile, new‐onset AF after TAVR is also common, which may be related to the operation itself. AF increases the risk of postoperative cardiogenic embolic events and increases the incidence of cardiovascular adverse events, cerebrovascular events, and mortality.^33^ In addition, bleeding events are also important events that should be widely concerned, which increases the difficulty of clinical antithrombotic therapy. Some risk factors, such as old age, frailty, falls, kidney disease, liver disease, malignant tumors, coagulation disorders, and antithrombotic therapy, may increase the risk of bleeding, which affects the patient's prognosis.^34^

Our study found that DOACs reduced the risk of all‐cause death in patients with AF after TAVR. In addition, subgroup analysis based on different follow‐up times showed that patients with longer follow‐up times benefited more significantly. The biggest difference between DOACs and VKAs is that DOACs can only inhibit one step in the coagulation process, while VKAs can prohibit multiple steps. Vitamin K is a cofactor of activation of coagulation factors ⅱ, ⅶ, ⅸ, ⅹ, and VKAs can reduce the synthesis of vitamin K‐dependent coagulation factors.^35^ The conditions under which VKAs are most effective are harsh. A variety of foods, particularly vegetables, and many drugs, such as inducers and inhibitors of hepatic P450 isoenzymes, may significantly alter the pharmacokinetics and pharmacodynamics of VKAs, increasing or decreasing the anticoagulant activity of VKAs unpredictably.^36^, ^37^ In addition, the use of VKAs requires frequent blood sampling to monitor INR, which poses significant challenges to patient compliance.^38^ The limitations of VKAs have also become the advantages of DOACs. The clinical trials of DOACs published in the past mostly excluded patients with valvular heart disease. Now, with the continuous maturity of TAVR technology, the data are expanding, and some randomized controlled trials of antithrombotic strategies after TAVR are also being conducted, which will provide new evidence for our conclusion.

The choice of antithrombotic strategy after TAVR has been a hotly debated issue in the field. A meta‐analysis by Dr. Ueyama and colleagues included five studies comparing the safety and efficacy of DOACs versus VKAs in TAVR patients with an indication for anticoagulation, and they found that the risk of all‐cause mortality, bleeding, and stroke was similar between DOACs and VKAs.^39^ With the publication of RCT studies on antithrombotic strategies after TAVR, most notably the ATLANTIS study and the ENVISAGE‐TAVI AF study, we have updated this topic. The ATLANTIS study investigated the feasibility of DOACs as an antithrombotic after TAVR compared to standard regimens.^17^ The ATLANTIS study showed that for all patients undergoing TAVR with or without an indication for oral anticoagulation, the efficacy of apixaban after TAVR was not superior to the current standard antithrombotic regimen in terms of net clinical benefit. The ENVISAGE‐TAVI AF study was designed to investigate the efficacy of edoxaban in patients with recurrent or new‐onset AF after TAVR.^18^ The primary endpoint event rate was not inferior to the VKAs group in the edoxaban group, but the incidence of major bleeding was higher than in the VKAs group, mainly due to more gastrointestinal bleeding in the edoxaban group. Compared with the previous meta‐analysis, we concluded that anticoagulation with DOACs in patients with TAVR combined with AF may be superior to VKAs, but we need more evidence to prove our point in the future, and the quest for the best antithrombotic treatment option after TAVR will continue.

Our meta‐analysis has several limitations. First, our study included two RCT, one CCT, and eight cohort studies. Although we adopted some quality evaluation methods, bias will inevitably occur, and more RCTs will be needed in the future to verify our conclusions. Second, some studies used antiplatelet drugs combined with anticoagulant therapy, while others did not. Due to limited data, we did not conduct a separate subgroup analysis. Also, we did not conduct a subgroup analysis of the patient population, so the heterogeneity of the patient population should be considered when interpreting the study results. Finally, due to the lack of data, we did not conduct a subgroup analysis of the types of DOACs and could not evaluate the individual category effect of each DOAC.

## 5 CONCLUSION

For patients with AF after TAVR, the use of DOACs may be superior to VKAs, and the benefit may be greater with longer follow‐up. The anticoagulant strategy for AF after TAVR is a valuable direction for future research.

## AUTHOR CONTRIBUTIONS

**Jie Yan**: Writing—original draft; methodology; software; visualization. **Ming Liu, Yu Zhang, and Danning Yang**: Data curation. **Fengshuang An**: Writing—reviewing and editing.

## CONFLICT OF INTEREST

The authors declare no conflict of interest.

## Supporting information

Supplementary information.

## Data Availability

All data were extracted from the included studies and all data involved were presented in Supporting Information: Materials.

## References

[1] Yi B , Zeng W , Lv L , Hua P . Changing epidemiology of calcific aortic valve disease: 30‐year trends of incidence, prevalence, and deaths across 204 countries and territories. Aging. 2021;13(9):12710‐12732. 33973531 10.18632/aging.202942 PMC8148466

[2] Yadgir S , Johnson CO , Aboyans V , et al. Global, regional, and national burden of calcific aortic valve and degenerative mitral valve diseases, 1990–2017. Circulation. 2020;141(21):1670‐1680. 32223336 10.1161/CIRCULATIONAHA.119.043391

[3] Ross J Jr. , Braunwald E . Aortic stenosis. Circulation. 1968;38(1 suppl):61‐67. 4894151 10.1161/01.cir.38.1s5.v-61

[4] Turina J , Hess O , Sepulcri F , Krayenbuehl HP . Spontaneous course of aortic valve disease. Eur Heart J. 1987;8(5):471‐483. 3609042 10.1093/oxfordjournals.eurheartj.a062307

[5] Cheitlin MD , Gertz EW , Brundage BH , Carlson CJ , Quash JA , Bode RS Jr. Rate of progression of severity of valvular aortic stenosis in the adult. Am Heart J. 1979;98(6):689‐700. 495418 10.1016/0002-8703(79)90465-4

[6] Leon MB , Smith CR , Mack M , et al. Transcatheter aortic‐valve implantation for aortic stenosis in patients who cannot undergo surgery. N Engl J Med. 2010;363(17):1597‐607. 20961243 10.1056/NEJMoa1008232

[7] Jørgensen TH , Thyregod HGH , Ihlemann N , et al. Eight‐year outcomes for patients with aortic valve stenosis at low surgical risk randomized to transcatheter vs. surgical aortic valve replacement. Eur Heart J. 2021;42(30):2912‐2919. 34179981 10.1093/eurheartj/ehab375 PMC8347457

[8] Tarantini G , Mojoli M , Urena M , Vahanian A . Atrial fibrillation in patients undergoing transcatheter aortic valve implantation: epidemiology, timing, predictors, and outcome. Eur Heart J. 2017;38(17):1285‐1293. 27744287 10.1093/eurheartj/ehw456

[9] Dawwas GK , Dietrich E , Cuker A , Barnes GD , Leonard CE , Lewis JD . Effectiveness and safety of direct oral anticoagulants versus warfarin in patients with valvular atrial fibrillation: a population‐based cohort study. Ann Intern Med. 2021;174(7):910‐919. 33780291 10.7326/M20-6194

[10] Molteni M , Cimminiello C . Warfarin and atrial fibrillation: from ideal to real the warfarin affaire. Thromb J. 2014;12(1):5. 24548437 10.1186/1477-9560-12-5 PMC3937065

[11] Connolly SJ , Ezekowitz MD , Yusuf S , et al. Dabigatran versus warfarin in patients with atrial fibrillation. N Engl J Med. 2009;361(12):1139‐1151. 19717844 10.1056/NEJMoa0905561

[12] Patel MR , Mahaffey KW , Garg J , et al. Rivaroxaban versus warfarin in nonvalvular atrial fibrillation. N Engl J Med. 2011;365(10):883‐891. 21830957 10.1056/NEJMoa1009638

[13] Granger CB , Alexander JH , Mcmurray JJV , et al. Apixaban versus warfarin in patients with atrial fibrillation. N Engl J Med. 2011;365(11):981‐992. 21870978 10.1056/NEJMoa1107039

[14] Giugliano RP , Ruff CT , Braunwald E , et al. Edoxaban versus warfarin in patients with atrial fibrillation. N Engl J Med. 2013;369(22):2093‐2104. 24251359 10.1056/NEJMoa1310907

[15] Hindricks G , Potpara T , Dagres N , et al. 2020 ESC Guidelines for the diagnosis and management of atrial fibrillation developed in collaboration with the European Association for Cardio‐Thoracic Surgery (EACTS): the task force for the diagnosis and management of atrial fibrillation of the European Society of Cardiology (ESC) developed with the special contribution of the European Heart Rhythm Association (EHRA) of the ESC. Eur Heart J. 2021;42(5):373‐498. 32860505 10.1093/eurheartj/ehaa612

[16] Vahanian A , Beyersdorf F , Praz F , et al. 2021 ESC/EACTS Guidelines for the management of valvular heart disease. Eur Heart J. 2022;43(7):561‐632. 34453165 10.1093/eurheartj/ehab395

[17] Montalescot G . Anti‐thrombotic strategy to lower all cardiovascular and neurologic ischemic and hemorrhagic events after trans‐aortic valve implantation for aortic stenosis—ATLANTIS. Paper presented at: American College of Cardiology Virtual Annual Scientific Session (ACC 2021); May 15, 2021. Available from: https://www.acc.org/education-and-meetings/image-and-slide-gallery/media-detail?id=f046941f6da6440985ba12ae6ec2e726

[18] Van Mieghem NM , Unverdorben M , Hengstenberg C , et al. Edoxaban versus vitamin K antagonist for atrial fibrillation after TAVR. N Engl J Med. 2021;385(23):2150‐2160. 34449183 10.1056/NEJMoa2111016

[19] Higgins JP , Altman DG , Gøtzsche PC , et al. The Cochrane Collaboration's tool for assessing risk of bias in randomised trials. BMJ. 2011;343:d5928. 22008217 10.1136/bmj.d5928 PMC3196245

[20] Slim K , Nini E , Forestier D , Kwiatkowski F , Panis Y , Chipponi J . Methodological index for non‐randomized studies (minors): development and validation of a new instrument. ANZ J Surg. 2003;73(9):712‐716. 12956787 10.1046/j.1445-2197.2003.02748.x

[21] Higgins JPT TJ , Chandler J , Cumpston M , Li T , Page MJ , Welch VA . Cochrane Handbook for Systematic Reviews of Interventions, Version 6.3 (Updated February 2022). Cochrane;2022. Available from: www.training.cochrane.org/handbook

[22] Mangner N , Crusius L , Haussig S , et al. Continued versus interrupted oral anticoagulation during transfemoral transcatheter aortic valve implantation and impact of postoperative anticoagulant management on outcome in patients with atrial fibrillation. Am J Cardiol. 2019;123(7):1134‐1141. 30658919 10.1016/j.amjcard.2018.12.042

[23] Butt JH , De Backer O , Olesen JB , et al. Vitamin K antagonists vs. direct oral anticoagulants after transcatheter aortic valve implantation in atrial fibrillation. Eur Heart J Cardiovasc Pharmacother. 2021;7(1):11‐19. 31665260 10.1093/ehjcvp/pvz064

[24] Kawashima H , Watanabe Y , Hioki H , et al. Direct oral anticoagulants versus vitamin K antagonists in patients with atrial fibrillation after TAVR. JACC Cardiovasc Interv. 2020;13(22):2587‐2597. 33129818 10.1016/j.jcin.2020.09.013

[25] Jochheim D , Barbanti M , Capretti G , et al. Oral anticoagulant type and outcomes after transcatheter aortic valve replacement. JACC Cardiovasc Interv. 2019;12(16):1566‐1576. 31202946 10.1016/j.jcin.2019.03.003

[26] Geis NA , Kiriakou C , Chorianopoulos E , Uhlmann L , Katus HA , Bekeredjian R . NOAC monotherapy in patients with concomitant indications for oral anticoagulation undergoing transcatheter aortic valve implantation. Clin Res Cardiol. 2018;107(9):799‐806. 29644411 10.1007/s00392-018-1247-x

[27] Tanawuttiwat T , Stebbins A , Marquis‐Gravel G , Vemulapalli S , Kosinski AS , Cheng A . Use of direct oral anticoagulant and outcomes in patients with atrial fibrillation after transcatheter aortic valve replacement: insights from the STS/ACC TVT registry. J Am Heart Assoc. 2022;11(1):e023561. 34970918 10.1161/JAHA.121.023561 PMC9075194

[28] Kalogeras K , Jabbour RJ , Ruparelia N , et al. Comparison of warfarin versus DOACs in patients with concomitant indication for oral anticoagulation undergoing TAVI; results from the ATLAS registry. J Thromb Thrombolysis. 2020;50(1):82‐89. 31605280 10.1007/s11239-019-01968-w

[29] Seeger J , Gonska B , Rodewald C , Rottbauer W , Wöhrle J . Apixaban in patients with atrial fibrillation after transfemoral aortic valve replacement. JACC Cardiovasc Interv. 2017;10(1):66‐74. 27916486 10.1016/j.jcin.2016.10.023

[30] Kosmidou I , Liu Y , Alu MC , et al. Antithrombotic therapy and cardiovascular outcomes after transcatheter aortic valve replacement in patients with atrial fibrillation. JACC Cardiovasc Interv. 2019;12(16):1580‐1589. 31439338 10.1016/j.jcin.2019.06.001

[31] Ranasinghe MP , Peter K , Mcfadyen JD . Thromboembolic and bleeding complications in transcatheter aortic valve implantation: insights on mechanisms, prophylaxis and therapy. J Clin Med. 2019;8(2):280. 30823621 10.3390/jcm8020280 PMC6406714

[32] Biviano AB , Nazif T , Dizon J , et al. Atrial fibrillation is associated with increased mortality in patients undergoing transcatheter aortic valve replacement: insights from the placement of aortic transcatheter valve (PARTNER) trial. Circulation Cardiovasc Interv. 2016;9(1):e002766. 10.1161/CIRCINTERVENTIONS.115.002766 PMC4704130 26733582

[33] Sannino A , Gargiulo G , Schiattarella GG , et al. A meta‐analysis of the impact of pre‐existing and new‐onset atrial fibrillation on clinical outcomes in patients undergoing transcatheter aortic valve implantation. EuroIntervention. 2016;12(8):e1047‐e1056. 26610809 10.4244/EIJY15M11_12

[34] Généreux P , Cohen DJ , Williams MR , et al. Bleeding complications after surgical aortic valve replacement compared with transcatheter aortic valve replacement: insights from the PARTNER I trial (placement of aortic transcatheter valve). JACC. 2014;63(11):1100‐1109. 24291283 10.1016/j.jacc.2013.10.058

[35] De Caterina R , Husted S , Wallentin L , et al. New oral anticoagulants in atrial fibrillation and acute coronary syndromes: ESC Working Group on Thrombosis‐Task Force on anticoagulants in heart disease position paper. JACC. 2012;59(16):1413‐1425. 22497820 10.1016/j.jacc.2012.02.008

[36] Holbrook AM . Systematic overview of warfarin and its drug and food interactions. Arch Intern Med. 2005;165(10):1095‐1106. 15911722 10.1001/archinte.165.10.1095

[37] Wittkowsky AK . Warfarin and other coumarin derivatives: pharmacokinetics, pharmacodynamics, and drug interactions. Semin Vasc Med. 2003;3(3):221‐230. 15199454 10.1055/s-2003-44457

[38] Hirsh J , Guyatt G , Albers GW , Harrington R , Schünemann HJ . Antithrombotic and thrombolytic therapy: American College of Chest Physicians Evidence‐based clinical practice guidelines (8th edition). Chest. 2008;133(6 suppl):110s‐112ss. 18574260 10.1378/chest.08-0652

[39] Ueyama H , Kuno T , Ando T , et al. Meta‐analysis comparing direct oral anticoagulants versus vitamin K antagonists after transcatheter aortic valve implantation. Am J Cardiol. 2020;125(7):1102‐1107. 31964500 10.1016/j.amjcard.2019.12.039

