# Supplementary material for: Comparison of efficacy and safety between VKAs and DOACs in patients with atrial fibrillation after transcatheter aortic valve replacement: A systematic review and meta‐analysis
Source: Clin Cardiol. 2022 Aug 28;45(10):1002–10. doi: 10.1002/clc.23909 (PMC9574758; doi:10.1002/clc.23909)
Supplement: Supplementary file 1 — Supplementary information. [file CLC-45-1002-s001.docx]

Supplementary Material

**1. Supplementary Figure 1: Quality evaluation of two RCT**

**
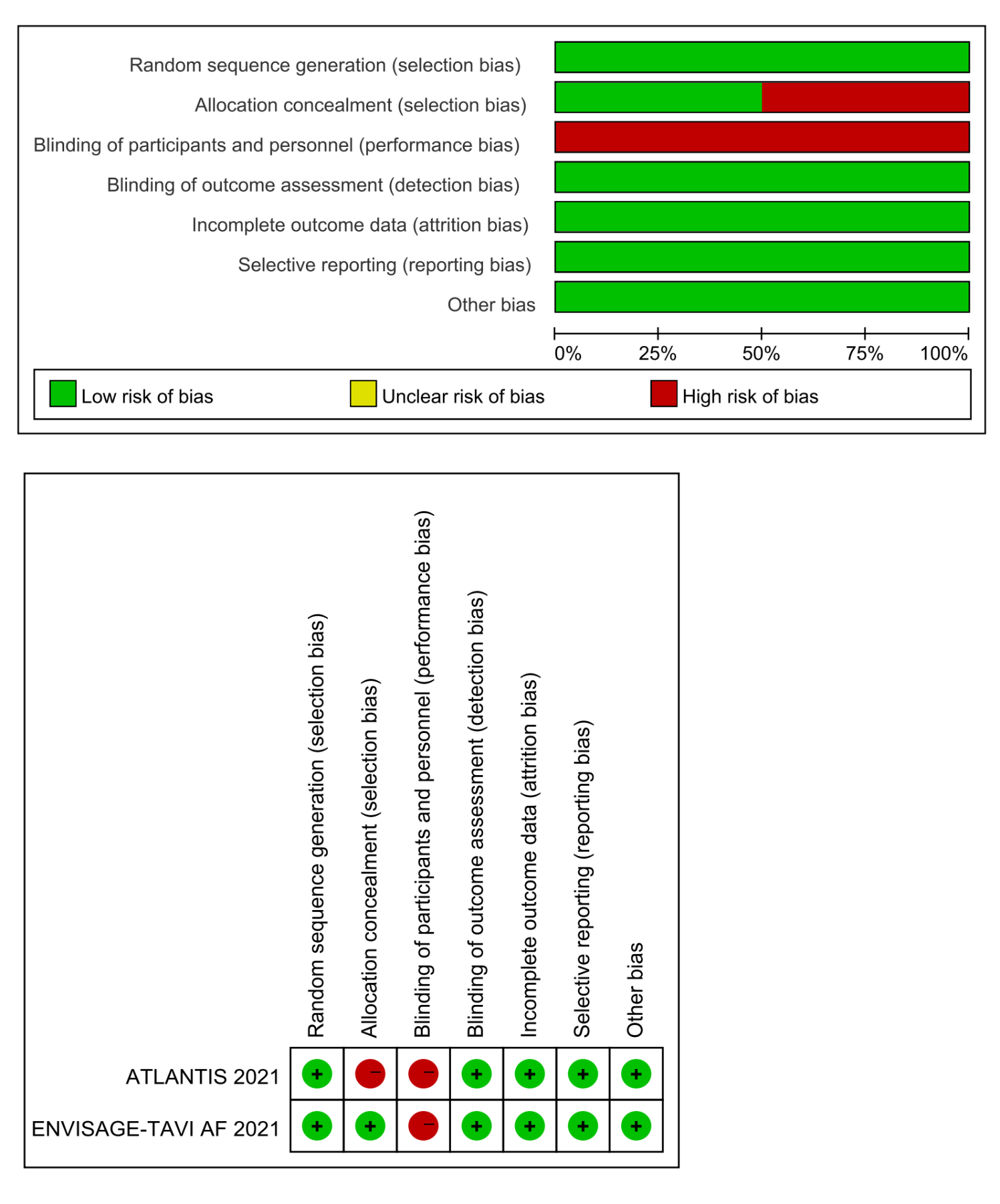
**

**2. Supplementary Table 1: Quality evaluation of one CCT**

| **study** | **A clearly stated aim** | **Inclusion of consecutive patients** | **Prospective collection of data** | **Endpoints appropriate to the aim of the study** | **Unbiased assessment of the study endpoint** | **Follow‐up period appropriate to the aim of the study** | **Loss to follow up less than 5%** | **Prospective calculation of the study size** | **An adequate control group** | **Contemporary groups** | **Baseline equivalence of groups** | **Adequate statistical analyses** | **Total Score** |
| --- | --- | --- | --- | --- | --- | --- | --- | --- | --- | --- | --- | --- | --- |
| Julia Seeger et al 2017 | 2 | 2 | 2 | 2 | 0 | 2 | 2 | 1 | 2 | 2 | 2 | 2 | 21 |

**3. Supplementary Table 2: Quality evaluation of eight observational studies (cohort studies)**

| **Study** | **Selection（score）** | | | | **Comparability（score）** | | **Outcome（score）** | | | **Total Score** |
| --- | --- | --- | --- | --- | --- | --- | --- | --- | --- | --- |
|  | **representativeness of the exposed cohort** | **Selection of the non-exposed cohort** | **Ascertainment of exposure** | **Demonstration that outcome of interest was not present at start of study** | **select the most important factor** | **any additional factor** | **Assessment of outcome** | **Follow-up period** | **Adequacy of follow up of cohorts** |  |
| Ioanna Kosmidou et al 2019 | 1 | 1 | 1 | 1 | 1 | 1 | 1 | 1 | 1 | 9 |
| Konstantinos Kalogeras et al 2019 | 1 | 1 | 1 | 1 | 1 | 0 | 1 | 1 | 0 | 7 |
| Tanyanan Tanawuttiwat et al 2022 | 1 | 1 | 1 | 1 | 1 | 1 | 1 | 1 | 1 | 9 |
| Nicolas A. Geis et al 2018 | 1 | 1 | 1 | 1 | 1 | 1 | 1 | 1 | 1 | 9 |
| Hideyuki Kawashima et al 2020 | 1 | 1 | 1 | 1 | 1 | 0 | 0 | 1 | 1 | 7 |
| David Jochheim et al 2019 | 1 | 1 | 1 | 1 | 1 | 0 | 1 | 1 | 0 | 7 |
| Jawad H. Butt et al 2021 | 1 | 1 | 1 | 1 | 1 | 1 | 1 | 1 | 0 | 8 |
| Norman Mangner et al 2019 | 1 | 1 | 1 | 1 | 1 | 0 | 1 | 1 | 0 | 7 |

**4. Funnel plot**

**Supplementary Figure 2a:** Funnel plot of all-cause death, detailing publication bias in the studies selected for analysis. Closed circles represent observed published studies. SE, standard error; RR, risk ratio.

**Supplementary Figure 2b:** Funnel plot of cardiovascular death, detailing publication bias in the studies selected for analysis. Closed circles represent observed published studies. SE, standard error; RR, risk ratio.

**Supplementary Figure 2c:** Funnel plot of stroke, detailing publication bias in the studies selected for analysis. Closed circles represent observed published studies. SE, standard error; RR, risk ratio.

**Supplementary Figure 2d:** Funnel plot of major and/or life-threatening, detailing publication bias in the studies selected for analysis. Closed circles represent observed published studies. SE, standard error; RR, risk ratio.


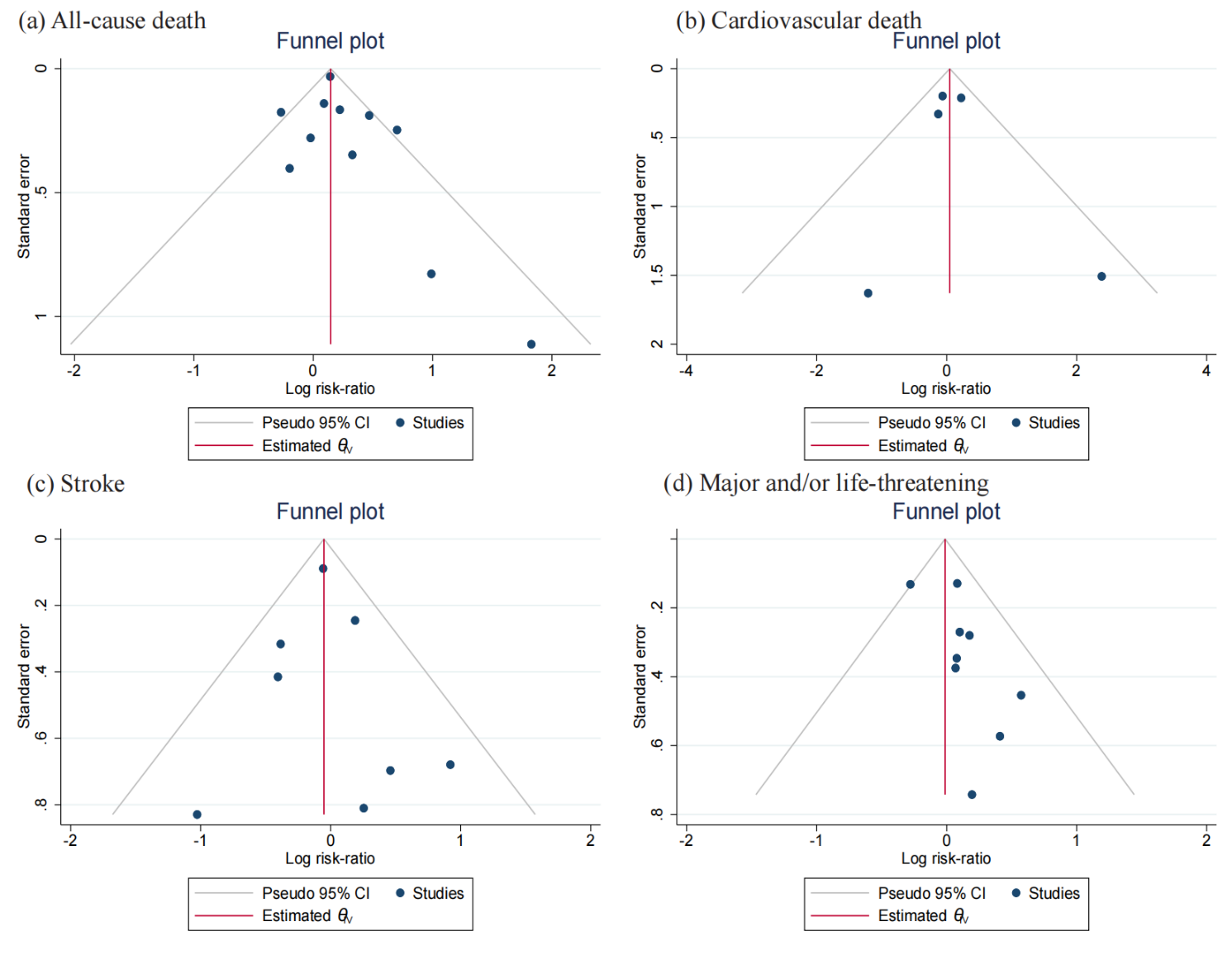


**5.Data**

All-cause deaths

| Study | Year | Eevent | Etotal | Cevent | Ctotal | E1event | C1event |
| --- | --- | --- | --- | --- | --- | --- | --- |
| ATLANTIS | 2021 | 23 | 228 | 23 | 223 | 205 | 200 |
| Butt et al | 2021 | 113 | 516 | 30 | 219 | 403 | 189 |
| ENVISAGE-TAVI AF | 2021 | 93 | 713 | 85 | 713 | 620 | 628 |
| Geis et al | 2018 | 11 | 172 | 12 | 154 | 161 | 142 |
| Jochheim et al | 2019 | 70 | 636 | 47 | 326 | 566 | 279 |
| Kalogeras et al | 2019 | 16 | 102 | 13 | 115 | 86 | 102 |
| Kosmidou et al | 2019 | 207 | 778 | 33 | 155 | 571 | 122 |
| OCEAN | 2020 | 36 | 176 | 23 | 227 | 140 | 204 |
| Mangner et al | 2019 | 4 | 117 | 1 | 182 | 113 | 181 |
| Seeger et al | 2017 | 5 | 131 | 2 | 141 | 126 | 139 |
| Tanawuttiwat et al | 2022 | 2367 | 13004 | 1284 | 8127 | 10637 | 6843 |

Cardiovascular death

| Study | Year | Eevent | Etotal | Cevent | Ctotal | E1event | C1event |
| --- | --- | --- | --- | --- | --- | --- | --- |
| ENVISAGE-TAVI AF | 2021 | 46 | 713 | 49 | 713 | 667 | 664 |
| Geis et al | 2018 | 0 | 172 | 1 | 154 | 172 | 153 |
| Jochheim et al | 2019 | 24 | 636 | 14 | 326 | 612 | 312 |
| Kosmidou et al | 2019 | 138 | 778 | 22 | 155 | 640 | 133 |
| Mangner et al | 2019 | 3 | 117 | 0 | 182 | 114 | 182 |

Stroke

| Study | Year | Eevent | Etotal | Cevent | Ctotal | E1event | C1event |
| --- | --- | --- | --- | --- | --- | --- | --- |
| ENVISAGE-TAVI AF | 2021 | 35 | 713 | 29 | 713 | 678 | 684 |
| Geis et al | 2018 | 2 | 172 | 5 | 154 | 170 | 149 |
| Jochheim et al | 2019 | 13 | 636 | 10 | 326 | 623 | 316 |
| Kosmidou et al | 2019 | 41 | 778 | 12 | 155 | 737 | 143 |
| Mangner et al | 2019 | 4 | 115 | 4 | 182 | 111 | 178 |
| OCEAN | 2020 | 3 | 176 | 3 | 227 | 173 | 224 |
| Seeger et al | 2017 | 7 | 131 | 3 | 141 | 124 | 138 |
| Tanawuttiwat et al 2022 | 2022 | 308 | 13004 | 204 | 8127 | 12696 | 7923 |

Major and/or life-threatening

| Study | Year | Eevent | Etotal | Cevent | Ctotal | E1event | C1event |
| --- | --- | --- | --- | --- | --- | --- | --- |
| ATLANTIS | 2021 | 26 | 228 | 23 | 223 | 202 | 200 |
| Butt et al | 2021 | 28 | 516 | 11 | 219 | 488 | 208 |
| Jochheim et al | 2019 | 146 | 636 | 69 | 326 | 490 | 257 |
| ENVISAGE-TAVI AF | 2021 | 87 | 713 | 115 | 713 | 626 | 598 |
| Kosmidou et al | 2019 | 43 | 778 | 8 | 155 | 735 | 147 |
| Seeger et al | 2017 | 7 | 131 | 5 | 141 | 124 | 136 |
| Geis et al | 2018 | 3 | 50 | 4 | 81 | 47 | 77 |
| Mangner et al | 2019 | 19 | 116 | 25 | 182 | 97 | 157 |
| OCEAN | 2020 | 11 | 176 | 8 | 227 | 165 | 219 |

Subgroup analysis

| Study | Year | Eevent | Etotal | Cevent | Ctotal | E1event | C1event | Follow-up |
| --- | --- | --- | --- | --- | --- | --- | --- | --- |
| Jochheim et al | 2019 | 7 | 636 | 7 | 626 | 629 | 619 | 1 month |
| Mangner et al | 2019 | 4 | 117 | 1 | 182 | 113 | 181 | 1 month |
| Seeger et al | 2017 | 5 | 131 | 2 | 141 | 126 | 139 | 1 month |
| ATLANTIS | 2021 | 23 | 228 | 23 | 223 | 205 | 200 | 1month-12month |
| Butt et al | 2021 | 54 | 516 | 15 | 219 | 462 | 204 | 1month-12month |
| Geis et al | 2018 | 11 | 172 | 12 | 154 | 161 | 142 | 1month-12month |
| Jochheim et al | 2019 | 70 | 636 | 47 | 326 | 566 | 279 | 1month-12month |
| Mangner et al | 2019 | 16 | 117 | 16 | 182 | 101 | 166 | 1month-12month |
| Seeger et al | 2017 | 6 | 50 | 19 | 81 | 44 | 62 | 1month-12month |
| Tanawuttiwat et al | 2022 | 2367 | 13004 | 1284 | 8127 | 10637 | 6843 | 1month-12month |
| Butt et al 2021 | 2021 | 113 | 516 | 30 | 219 | 403 | 189 | >12month |
| Kalogeras et al 2019 | 2019 | 16 | 102 | 13 | 115 | 86 | 102 | >12month |
| OCEAN | 2020 | 41 | 176 | 23 | 227 | 135 | 204 | >12month |
| ENVISAGE-TAVI AF | 2021 | 93 | 713 | 85 | 713 | 620 | 628 | >12month |
